# Longitudinal predictors of weapon involvement in middle adolescence: Evidence from the UK Millennium Cohort Study

**DOI:** 10.1002/ab.22049

**Published:** 2022-08-17

**Authors:** Aase Villadsen, Emla Fitzsimons

**Affiliations:** ^1^ Centre for Longitudinal Studies University College London London UK

**Keywords:** adolescence, longitudinal data, Millennium Cohort Study, weapon carrying, weapon involvement, weapon use

## Abstract

This study uses longitudinal data from the UK Millennium Cohort Study (*N* = 13,277) to examine the childhood and early adolescence factors that predict weapon involvement in middle adolescence, which in this study is exemplified by having carried or used a weapon. It finds that childhood experiences of low family income and domestic abuse between parents predict weapon involvement at age 17 years. Other predictors include childhood externalizing problems and self‐harm in early adolescence. Further early adolescent behaviors and experiences that predict weapon involvement are own substance use, peer substance use, school exclusion, and high levels of electronic gaming. These findings provide concrete areas for targeting risk factors both in childhood and the early adolescent period, with an indication that early intervention and prevention are likely to reduce the need for later action.

## 1 INTRODUCTION

Adolescence is a developmental stage characterized by biological and environmental changes that influence risk‐taking behaviors, including increased involvement in criminal and antisocial activities (Steinberg & Morris, 2001). The age distribution in offending behavior is well‐established; rates are low in childhood, increase dramatically from early adolescence with a peak in late adolescence, followed by a steep decline in the late teens and early 20, and a more steady decline through adulthood (Farrington, 1986; MacLeod et al., 2012). The surge in risk‐taking behaviors in adolescence is driven by several factors, including neurobiological, psychological, and social. The brain undergoes significant neurobiological development in adolescence, triggering changes to the socioemotional system, specifically in relation to reward‐seeking fueled by the brain's dopaminergic system, and particularly in the presence of peers (Blakemore & Choudhury, 2006; Steinberg, 2008). Psychologically, adolescents have not yet attained adult cognitive function in self‐regulation and are therefore not fully able to inhibit inappropriate behavioral or emotional responses (Luna, 2009; Tottenham et al., 2011). As for social contexts, adolescence is a time when individuals become more independent from their families and spend increasing time with peers who become a significant source of influence (Prinstein & Dodge, 2008; Steinberg & Silverberg, 1986).

Despite a higher prevalence of offending behavior being somewhat normative in adolescence, with the tendency for behaviors to be mostly adolescence‐limited (Jolliffe et al., 2017), these behaviors are nonetheless a concern as they pose a risk of onward development of criminal behavior (Moffitt, 2006), and there is evidence that weapon carrying in adolescence is highly predictive of similar adult behaviors (Wallace, 2017). Importantly, adolescent offending, particularly in relation to serious and violent crimes involving weapons, can cause significant harm to others.

Offenses classified as “violence against the person” account for the largest volume of youth offenses in the United Kingdom, and have been rising in recent years (Youth Justice Board/Ministry of Justice, 2021), with 31% of all proven offenses being classified as such in 2020, a significant rise from 20% in 2010. Moreover, the number of knife or other weapon offenses committed by youths was a stark 46% higher in 2020 than just 5 years previously. The majority (72%) of violence against the person offenses was committed by young people aged 15–17, which is further evidence that the late adolescent period is a particularly acute one for offending. Consistent with these crime statistics, there is also evidence that hospital episodes for assault by a sharp object involving 16–18‐year‐olds in England rose from 340 in 2015 to 585 in 2020 (NHS Digital, 2020).

As the alarming statistics highlight, reducing offending involving weapons in young people remains an important task for policymakers and practitioners. The UK Government has responded with the introduction of the Offensive Weapons Act 2019, which introduced new offenses in relation to a wide range of weapons, including the prohibition of possessing weapons in private. Another response has been the introduction of Violence Prevention Units take a public health approach by addressing the root causes of violent crime and bringing together multiple organizations across local communities to address the risk factors through evidence‐based early intervention and prevention with a focus on generating a long‐term as well as a short‐term solution (Home Office, 2020). Such an approach has long been endorsed by developmental criminologists, who have emphasized the importance of understanding the underlying factors and mechanisms driving offending behaviors, especially early factors much before offending behaviors, which may provide opportunities for early prevention and intervention (Sutton et al., 2006).

A widely used framework for understanding factors influencing child and human development more broadly is Bronfenbrenner's ecological systems theory (Bronfenbrenner, 1979). This highlights the multicausal nature of behavioral outcomes, and in early development, the so‐called “microsystem” is considered especially important as this involves the groups and institutions in the developing child's immediate environment with whom it has a direct contact and regular interaction. This is reflected in studies that have shown a multiple range of factors to influence adolescent offending, including individual factors, familial factors, such as family socioeconomic and psychosocial family risks, and other environmental influences, such as peers and schools (Case & Haines, 2009). Given the centrality of the childhood period to later offending, longitudinal data are especially useful for understanding the early antecedents of offending behaviors, including weapon offenses (Huesmann et al., 2021; Salo et al., 2021).

Using rich and nationally representative longitudinal data from the UK Millennium Cohort Study (MCS) on young people, their families, and wider social contexts, the primary aim of the current study is to understand the main drivers of weapon involvement, which in this study is exemplified by weapon carrying and/or use at age 17 years. Adopting the Bronfenbrenner model and multicausal approach, we hypothesized several aspects across the social ecology of the child to be associated with weapon involvement in late adolescence. By examining a range of factors across children's formative years as well as in early adolescence, in a current cohort of adolescents, this study provides important up‐to‐date evidence that can help inform policy to reduce serious violence.

## 2 METHOD

### 2.1 Data

The MCS is the UK nationally representative birth cohort study following an initial sample of over 19,000 individuals born around the millennium (September 2000–January 2002) (Joshi & Fitzsimons, 2016). The initial survey was at age 9 months, with follow‐ups at ages 3, 5, 7, 11, 14, and 17 years. This longitudinal study is highly multidisciplinary with detailed information collected on individuals (participants) and their families. These include areas such as socioeconomic circumstances, family structure, childrearing environment, and parental characteristics, as well as social, cognitive and behavioral development, and the mental and physical health of participants at key life stages. In the initial survey, interviews with parents were solely relied on, but from as early as age 3 participants were increasingly involved (initially via objective cognitive assessments and physical measurements), and have been reporting increasingly on their experiences, behaviors, and activities since age 7 years. This included, at age 17 years, information about their involvement in a range of offending behaviors, including weapon carrying and use.

### 2.2 Measures

#### 2.2.1 Weapon involvement

At age 17 years, participants were asked: “In the last 12 months have you carried a knife or other weapon? For your own protection, because someone else asked you to or in case you get into a fight.” They were also asked: “In the past year have you hit someone with or used a weapon?” Participants who had either carried or used a weapon were in this study identified as having engaged in weapon involvement.

#### 2.2.2 Predictor variables

Potential predictors of weapon involvement included *individual characteristics* (sex, age in months, whether or not eldest child in household, ethnicity), *socioeconomic background* (household income age 9 months to 11 years), *family environment* (breastfeeding, maternal smoking during pregnancy, parent–child relationship at age 3 years, parent mental health at age 9 months to 11 years, domestic abuse between parents at age 9 months to 11 years, parent recreational drug use at age 3–14 years, single parent status at age 9 months to 11 years), *school factors* (academic achievement at age 16 years, school exclusion at age 14 years, truancy at age 14 years), and *peer factors* (amount of time spent with peers, victim of bullying, peer substance use, all at age 14 years). In addition, prior *behavioral factors* were examined: child externalizing and internalizing problems (age 3–11 years), and self‐harm, social media use, gaming, and substance use (all at age 14 years). Further details on the measurement of predictor variables are available in Supporting Information: Table S1.

### 2.3 Analyses

All analyses were carried out using STATA version 16 (StataCorp, 2019). Both multiple imputation and weighing were used to deal with missing data arising mainly from attrition. Multiple imputation is an efficient method for replicating population estimates in longitudinal data when sections of data are missing (Mostafa et al., 2021), under the assumption that there is a pattern to the missingness and that this can be predicted by the observed data (Little & Rubin, 2002). A rule of thumb when imputing missing data is to impute up to 50% of missing data, as imputing above this threshold can reduce accuracy and can bias estimates (Mishra & Khare, 2014). Therefore, missing data were imputed back to the age 11 survey, creating 30 imputed data sets through multiple chained equations. To improve the accuracy of imputed values and estimates, several auxiliary variables that were not part of the substantial analysis were used in imputations (Von Hippel & Lynch, 2013), including child cognition and school readiness at age 3 years, observed positive and negative child behaviors at age 3 years, and antisocial behaviors at age 11 years. To further adjust for attrition or missingness between the initial sweep and age 11 years, inverse probability weights were used, and weights were also adjusted for the complex sampling design of the initial MCS survey (Mostafa, 2015). The final sample used in the current analyses consists of 13,277 participants.

For the substantive analyses, weapon involvement was predicted using multivariate logistic regression using a wide range of potential predictors spanning different domains of participants' lives and all measured before age 17 years. The MCS is extremely rich in the information collected, and where variables measuring similar concepts were available, only one was included in the prediction model. For instance, household income was chosen over other measures of socioeconomic status as it was the strongest predictor in this domain. Variables were entered incrementally in blocks, to permit the study of more distal aspects such as family circumstance and environment in childhood, before examining later factors in adolescence such as substance use, peer, and school factors. In additional analyses, regression models were estimated separately for males and females in order to examine whether risk factors for weapon involvement differed by sex, and analyses were followed up with models that included interaction terms between sex and predictor variables.

## 3 RESULTS

Of the 13,277 participants, 52% were males, 84% were of White ethnic origin, around 40% had at least one parent with a university degree or higher degree and nearly a quarter (23%) received free school meals in primary school, which indicates a low household income. The prevalence of weapon involvement in the past year at age 17 years was 6.4% (95% confidence interval [CI] = 5.5–7.3). Further sample characteristics and descriptive statistics of study measures are shown in Table 1.

**Table 1:** Sample characteristics and descriptive statistics of study measures (*N* = 13,277)

	Percent or mean	SD
Individual characteristics		
Male	51.6%	
Eldest child in the household	42.5%	
Cohort member age in months at age 17 survey	17.2 years	0.34
Ethnicity		
White	84.4%	
Mixed	3.5%	
Indian	2.0%	
Pakistani and Bangladeshi	5.0%	
Black or Black British	3.5%	
Other ethnic groups (incl. Chinese)	1.5%	
Family socioeconomics		
Household income weekly (average 9 months to age 11 years)	£365.2	178.2
Quintiles		
20% lowest	£150.2	22.9
20%–40%	£233.3	28.1
40%–60%	£330.3	29.7
60%–80%	£438.3	36.1
80%–100% highest	£636.5	111.0
Highest education in the household		
No formal qualifications	10.4%	
NVQ 1	6.6%	
NVQ 2	27.0%	
NVQ 3	16.3%	
NVQ 4	33.2%	
NVQ 5	6.5%	
Free school meals at age 3 or 5 years	22.6%	
UK country		
England	82.3%	
Wales	4.9%	
Scotland	8.7%	
Northern Ireland	4.1%	
Family environment		
Breastfed	66.2%	
Mother smoked during pregnancy	17.4%	
Parent–child relationship (parent‐reported) age 3 years	48.5	7.3
Main parent mental health problems (9 months to 11 years)	3.9	3.3
Domestic abuse between parents (9 months to 11 years)	22.3%	
Main parent used recreational drugs (age 3, 5, or 14 years)	8.6%	
Ever single parent between 9 months and 11 years	38.6%	
Mental health		
Childhood externalizing problems (age 3–11 years)	5.5	3.2
Childhood internalizing problems (age 3–11 years)	3.0	2.3
Self‐harmed in the past year (age 14 years)	15.7%	
Substance use at age 14 years		
Binge drinking, regular smoking, trying cannabis/drugs		
None of these	87.1%	
One type of substance	8.1%	
Two or three types of substances	4.8%	
Social media and gaming at age 14 years		
Social media time use		
None	7.9%	
Less than half an hour	12.0%	
Half an hour to less than 1 h	14.0%	
1 h to less than 2 h	16.7%	
2 h to less than 3 h	15.3%	
3 h to less than 5 h	14.1%	
5 h to less than 7 h	9.9%	
7 h or more	10.1%	
Computer/electronic gaming time use		
None	17.6%	
Less than half an hour	13.0%	
Half an hour to less than 1 h	11.2%	
1 h to less than 2 h	15.3%	
2 h to less than 3 h	13.9%	
3 h to less than 5 h	13.3%	
5 h to less than 7 h	7.3%	
7 h or more	8.3%	
School factors		
Academic achievement at age 16 years (give GCSEs Grade C or above)	56.6%	
School exclusion between age 11 and 14 years	7.0%	
Persistent truancy (more than just the once) past year at age 14 years	5.5%	
Peer factors at age 14 years		
Spending time with friends in leisure time on most days	38.8%	
Victim of peer bullying	49.0%	
Peer substance use (alcohol, smoking, drugs)		
No substance use	37.0%	
One type of substance	26.8%	
Two or three types of substances	36.2%	
Prevalance of weapon involvement at age 17 years	6.4% (95% CI = 5.5–7.3)	

Abbreviation: CI, confidence interval.

### 3.1 Predictors of weapon involvement

Results of regression predicting weapon involvement at age 17 years are shown in Table 2. Model 1 includes individual characteristics and family socioeconomic circumstances and family environment. We see that males had a higher risk of engaging in weapon involvement (odds ratio [OR] = 2.36; 95% CI = 1.86–3.02). Those growing up in households with low income (lowest quintile), as measured from age 9 months to 11 years, were more likely to use a weapon (OR = 1.73; 95% CI = 1.03–2.90) compared to those from high‐income households (highest quintile). Domestic abuse between parents during childhood (9 months to 11 years) was also a significant risk factor (OR = 1.36; 95% CI = 1.04–1.80). In Model 2, parental‐reported mental health in childhood and adolescence are added to the regression. This shows that a one standard deviation increase in externalizing problems in childhood (ages 3–11) was associated with higher odds of weapon involvement at age 17 years (OR = 1.29; 95% CI = 1.10–1.52), and self‐harm in early adolescence at age 14 years increased the odds over twofold (OR = 2.09; 95% CI = 1.56–2.70). Finally in Model 3, a range of participants' experiences and behaviors at age 14 years in terms of substance use, school, and peers are included. Results show that substance use at age 14 years was a significant predictor, especially the use of multiple types of substances (OR = 2.08; 95% CI = 1.21–3.58). Independent of own substance use, having peers at age 14 years who used multiple substances increased the risk of weapon involvement at age 17 years (OR = 1.99; 95% CI = 1.39–2.86). Another significant predictor at age 14 years was school exclusion (OR = 1.77; 95% CI = 1.08–2.93), and the risk was increased for those who spent a lot of time (7 h or more) on electronic or computer gaming (OR = 1.75; 95% CI = 1.01–3.02).

**Table 2:** Results of multivariate logistic regression predicting weapon involvement at age 17 years

	Model 1		Model 2		Model 3	
	OR	95% CI	OR	95% CI	OR	95% CI
Individual characteristics						
Male	**2.36** ***	1.86–3.02	**2.53** ***	1.97–3.25	**2.10** ***	1.50–2.96
Eldest child in the household	0.93	0.70–1.23	0.95	0.71–1.26	1.01	0.76–1.35
Cohort member age in months at age 17 survey	1.00	0.96–1.03	1.00	0.97–1.03	0.99	0.95–1.02
Ethnicity (ref. White)						
Mixed	0.91	0.45–1.83	0.93	0.46–1.87	0.98	0.47–2.07
Indian	0.78	0.30–2.02	0.85	0.33–2.22	1.27	0.50–3.23
Pakistani and Bangladeshi	0.71	0.45–1.12	0.84	0.52–1.35	1.25	0.77–2.03
Black or Black British	0.76	0.36–1.61	0.87	0.41–1.84	1.13	0.54–2.34
Other ethnic groups (incl. Chinese)	0.55	0.12–2.58	0.62	0.13–2.95	0.86	0.18–4.18
Family socioeconomics						
Household income weekly (average 9 months to age 11 years) (ref. 80%–100% highest)						
20% lowest	**1.73** *	1.03–2.90	1.57^+^	0.93–2.66	1.32	0.78–2.25
20%–40%	1.57^+^	0.99– 2.47	1.46	0.92–2.31	1.30	0.82–2.05
40%–60%	1.23	0.85–1.79	1.17	0.80–1.72	1.11	0.76–1.64
60%–80% highest	1.19	0.78–1.81	1.15	0.76–1.76	1.12	0.74–1.71
Family environment						
Breastfed	0.89	0.69–1.15	0.90	0.70–1.16	0.94	0.72–1.22
Mother smoked during pregnancy	1.25	0.91–1.71	1.20	0.88–1.64	1.06	0.76–1.48
Parent–child relationship (parent‐reported) age 3 years^a^	0.95	0.84–1.09	1.04	0.90–1.21	1.02	0.88–1.18
Main parent mental health problems (9 months to 11 years)^a^	1.06	0.92–1.21	1.04	0.90–1.20	1.04	0.90–1.21
Domestic abuse between parents (9 months to 11 years)	**1.36** *	1.04–1.80	**1.34** *	1.02–1.77	1.24	0.92–1.66
Main parent used recreational drugs (age 3, 5 or 14 years)	1.47+	0.95–2.28	1.41	0.90–2.21	1.17	0.72–1.89
Single parent ever between 9 months and 11 years	1.29	0.95–1.77	1.26	0.92–1.73	1.08	0.77–1.50
Mental health						
Childhood externalizing problems (age 3–11 years)^a^			**1.29** ^**^	1.10–1.52	1.10	0.92–1.30
Childhood internalizing problems (age 3–11 years)^a^			0.87	0.74–1.03	0.92	0.77–1.10
Age 14 years: Self‐harmed in the past year			**2.09** ^***^	1.56–2.79	1.39^+^	0.99–1.93
Substance use at age 14 years						
Binge drinking, regular smoking, trying cannabis/drugs (ref. none of these)						
One type of substance					**1.49** *	1.02–2.18
Two or three types of substances					**2.08** ^**^	1.21–3.58
Social media and gaming at age 14 years						
Social media time use^b^					1.09	0.63–1.87
Computer/electronic gaming time use^b^					**1.75** *	1.01–3.02
School factors						
Academic achievement at age 16 years (five GCSEs Grade C or above)					0.81	0.61–1.07
School exclusion between age 11 and 14 years					**1.77** *	1.08–2.93
Persistent truancy (more than just the once) in the past year at age 14 years					1.42	0.89–2.26
Peer factors at age 14 years						
Spending time with friends in leisure time on most days					1.15	0.89–1.49
Victim of peer bullying					1.13	0.88–1.45
Peer substance use (alcohol, smoking, drugs) (ref. no substance use)						
One type of substance					1.36	0.94–1.97
Two or three types of substances					**1.99** ^***^	1.39–2.86

Abbreviations: CI, confidence interval; OR, odds ratio.

^a^ This predictor variable is standardized (*z*‐score), meaning that the OR coefficient is for one standard deviation increase in the predictor.

^b^ This predictor variable is a ridit score, which is a transformation of ordinal scale responses (hourly time‐use bands) into a continuous measure. The OR coefficient corresponds to differences between those with the highest time use (7 h or more) compared to those with the lowest (no time spent).

*** *p* < .001

** *p* < .01

* *p* < .05

^+^ *p* < .10.

From Models 1 to 3, we see that the association between earlier childhood experiences and mental health weakened or disappeared once age 14 experiences and behaviors were included. This suggests that these early adolescence experiences are an important channel (mediator) through which early life experiences matter for later outcomes. Additional analyses that examined sex differences showed that risk factors for weapon involvement at age 17 years were similar for males and females (shown in Supporting Information: Tables S2 and S3). These were followed up by analyses that included an interaction term between sex and the significant predictors from the main results, none of which showed a significant interaction (Supporting Information: Table S4).

In other additional analyses, we dropped the externalizing measure from the models. Because childhood externalizing is included in Models 2 and 3, which include an item on aggression (“Often fights with other children or bullies them”), this may lead to overcontrolling and result in estimates for other independent variables being conservative. Results of models without externalizing problems are reported in Supporting Information: Table S5, showing that the same independent variables were significant predictors and that the magnitude of estimates was only slightly higher.

## 4 DISCUSSION

This study examined weapon involvement at age 17 years. Family experiences in childhood that predicted weapon involvement at age 17 years included low family income and domestic abuse between parents. Other predictive factors were childhood externalizing problems and self‐harm in early adolescence. Further early adolescent behaviors and experiences that predicted age 17 years weapon involvement were: own substance use, peer substance use, school exclusion, and a high level of gaming. Risk factors were similar for males and females. Results are discussed in terms of consistency with previous research and implications for policy and prevention.

### 4.1 Sex

One of the most consistent findings in relation to weapon involvement was the much higher prevalence among males than females, which has been shown in numerous previous studies (DeLisi & Vaughn, 2016), and reflected in official statistics (Ministry of Justice, 2020). This suggests that biological sex is a potential driver in offending and is supportive of evolutionary approaches to understanding crime (Durrant, 2019). However, the narrowing of the gap in offending between males and females over time highlights that social aspects of sex also play a role, as women's liberation and movement toward equality have likely meant that differences in many social behaviors and norms have also narrowed (Estrada et al., 2016). In terms of risk factors for weapon involvement, we found these to be similar for males and females, which is consistent with a recent summary of the evidence, which concluded that no risk factor is a significant predictor for just one sex, but some factors can be slightly more or less predictive for one sex (Her Majesty's Prison & Probation Service, 2019). The uniformity of risk factors for males and females is important for prevention and intervention, suggesting that sex‐specific programmes in terms of targeting risks are not necessarily required. However, there may still be a need for different approaches in relation to engagement and implementation.

### 4.2 Ethnicity

We found no evidence of ethnic minority groups reporting different rates of weapon involvement than those of white origin. Other studies relying on self‐reported offending have tended to find very few ethnic differences, while official crime statistics show higher rates among ethnic minorities (Ministry of Justice, 2019). Bias in the criminal justice system against ethnic minorities has been highlighted as a possible explanation (Phillips & Bowling, 2017). Ethnicity in relation to offending is a complex matter as different patterns may exist for different types of offenses, and as also reflected in official offending statistics it is useful to consider ethnic groups separately rather than under a combined Black, Asian, and minority ethnic (BAME) heading as there is much heterogeneity between groups. There is a plan to link the Police National Computer data, which stores criminal record information across the United Kingdom, to the MCS. This linkage will enable further research on criminal convictions in the MCS and ethnic disparities while drawing on other information in this rich longitudinal data set.

### 4.3 Family circumstances

Our finding that low household income during childhood was linked to later weapon involvement reflects a well‐established pattern in the criminological literature (Wright et al., 1999). However, this relationship dissipated when including other psychosocial aspects of the family environment, indicating that it is through these more proximal family mechanisms that socioeconomic status influences adolescent offending. Previous research has provided support for a reciprocal relationship between socioeconomic status and family processes, that is, these mutually affect each other (Conger et al., 2010). In terms of implications for policy, strategies are needed that directly address low income while also targeting the family environment in which children and adolescents develop.

A significant family environment risk factor for weapon involvement was domestic abuse between parents, which has been identified also in numerous previous studies as detrimental to child developmental outcomes such as conduct problems (Evans et al., 2008) and later violence (Payne et al., 2011). This highlights the importance of early identification of domestic violence, such as through routine screening by midwives and other healthcare professionals (Bacchus et al., 2004), or novel digital approaches such as online screening tools and hidden mobile reporting apps (Jeyaraman & Chandan, 2020), in addition to the need for investment in an evidence‐based intervention that reduces domestic violence such as through domestic violence perpetrator programmes (Lilley‐Walker et al., 2018). Such strategies may be an effective approach for reducing serious violence later in adolescence and beyond.

### 4.4 Mental health

Our analysis showed that a high level of externalizing symptoms (conduct problems and hyperactivity) in childhood was related to weapon involvement at age 17 years, and this association was potentially mediated through a range of experiences and behaviors at age 14 years. We also found that self‐harm at age 14 years was significantly related to weapon involvement at age 17 years, although the relationship was reduced when including other age 14 behaviors such as own and peer substance use, school exclusion, and gaming, suggesting that these are potential mediators. While the link between offending behaviors and externalizing problems is well‐established (Farrington & Loeber, 1990; Fergusson et al., 2005), the association with internalizing symptoms such as self‐harm has been less well researched. However, there is evidence that internalizing behaviors in recent generations of adolescents have a different association with antisocial behavior compared to past generations. This was examined in a study using our MCS sample at age 14 years, where it was found that antisocial behaviors were a stronger predictor of concurrent depressive symptoms compared to a cohort of 14‐year‐olds 10 years previously; it was also found that depressive symptoms were a weaker predictor of antisocial behaviors in the younger MCS sample, suggesting that these behaviors had become more concentrated in those with mental health difficulties (Gage & Patalay, 2021). Together, these results indicate that mental health may be an important avenue for intervention that may reduce weapon involvement and other offending behaviors in young people.

### 4.5 Substance use

Participants' use of substances had a close link with weapon involvement at age 17 years. The findings are consistent with previous research and extremely well established in the literature (White, 2016). Mental health tends to be related to substance use (Gray & Squeglia, 2018), also previously shown in the current MCS sample (Gage & Patalay, 2021), and it may be a driver of the association between substance use and offending. However, the association with substance use in our analysis remained after controlling for childhood and adolescent mental health.

In addition to own substance use, peer substance use at age 14 years was associated with weapon involvement at age 17 years. The importance of peers has also been demonstrated in a wealth of previous research (Dishion & Tipsord, 2011). However, this association may reflect the direct influence of peers or simply selection effects whereby adolescents chose peers with similar interests, attitudes, and behaviors (also referred to as homophily). Previous studies tend to show that most peer effects are due to selection (Kandel, 1978; Vitaro et al., 2015). In our study, while the association between own substance use and weapon involvement was reduced somewhat when peer substance use was controlled for, both remained significantly associated with this outcome. In terms of policy implications, targeting substance use in adolescence may be an important element to help reduce weapon involvement and other offenses. While some interventions are targeted at the individual, others focus specifically on resistance to peer pressure, with evidence of effectiveness (Griffin & Botvin, 2010). It is also worth considering that the association with peer substance use may be explained in part by other peer behaviors such as delinquency and gang involvement; however, such information was not available in our data. Such possible peer contagion effect of antisocial behaviors suggests that interventions that successfully reduce these behaviors in young people may have much wider effects by also reaching the peer networks of those receiving an intervention.

### 4.6 School exclusion

We found that being excluded from school on a temporary or permanent basis between the ages of 11 and 14 was related to weapon involvement at age 17 years. While we do not claim our estimates are causal, they are nonetheless robust in controlling for the potentially confounding effects of a wide array of individual and family background factors and childhood externalizing problems, as well as other experiences and behaviors at age 14 years—unlike previous UK studies on exclusion (Ministry of Justice, 2018; Smith & Wynne‐McHardy, 2019). Findings highlight the importance of early intervention to prevent school exclusion.

### 4.7 Gaming

Our finding that spending a lot of time on computer/electronic gaming at age 14 years was associated with weapon involvement at age 17 years warrants discussion. Results are consistent with a meta‐analysis of 98 previous studies showing that exposure specifically to violent video games is associated with an increase in aggression and a decrease in prosocial behaviors, whereas prosocial video games have the opposite association (Greitemeyer & Mugge, 2014). However, it is important to highlight that the nature of video gaming was not observed in our data and therefore we were not able to separate the content from the time spent on gaming. Assuming that much of the excessive gaming involved violence, it is of course plausible that those who play violent games excessively are a selective group, with preferences that also make them more likely to engage in offending behaviors. However, some previous research has indicated a potential causal link between violent video gaming and weapon use, as shown in a randomized controlled trial where children's exposure to violent video games increased dangerous behaviors around firearms, such as picking up, handling, pointing, and squeezing the trigger of a real (disabled) gun (Chang & Bushman, 2019). The effect of video gaming on weapon involvement is an important area for future research, given the large increases in gaming in recent years (Clement, 2021). It is interesting that prosocial games have been found to be associated with prosocial behaviors—including in experimental studies (Gentile et al., 2009)—which may be an opportunity for intervention to increase positive child behaviors, as well as being informative for parents. It is also worth noting the argument that the computer and internet revolution has led to young people spending an increasing amount of time at home, and that overall this has been a driver for a decrease in youth offending rates (Aebi & Linde, 2014).

### 4.8 Strengths and limitations of the study

The strengths of this study included the large sample of participants representing the whole of the United Kingdom. The longitudinal design, with follow‐ups from birth and at developmentally important timepoints through childhood, offered a rich set of variables to examine as predictors of weapon involvement. The use of multiple imputations and weighting adjusted for the differential attrition of participants over time and is likely to provide more accurate estimates than in other similar studies. Of course, attrition from the longitudinal study over time is still a limitation and it is always optimal to have complete observed data from all participants. In terms of other limitations, although care was taken to control for a wide range of predictor variables, all measured before the outcome at age 17 years, thereby mitigating issues around omitted variable bias and reverse causality, we cannot claim that the factors found to be associated with weapon involvement have a causal effect. One omitted but potentially important factor that may account for the association between some of the family environment and family socioeconomic factors is genetics. With the recent availability of genetic information in the MCS sample, this is an area for future research.

## 5 CONCLUSION

In conclusion, this study showed that multiple factors were associated with weapon involvement in middle adolescence, which suggests a need for a range of strategies to target these core areas. Strategies in childhood should target low family income, domestic abuse between parents and child conduct problems. In early adolescence, the focus should be on adolescent mental health, substance use, peer substance use and school exclusion. Policies to help improve children's lives earlier on may reduce the need for later intervention, as risk factors identified in childhood appear to link weapon involvement to risk factors in the early teenage years.

## CONFLICT OF INTEREST

The authors declare no conflict of interest.

## Supporting information

Supporting information.

## Data Availability

The data that support the findings of this study are openly available in UK Data Archive at https://ukdataservice.ac.uk, reference number https://doi.org/10.5255/UKDA-SN-8756-1. Data are available to researchers from the UK Data Service (https://beta.ukdataservice.ac.uk/datacatalogue/series/series?id=2000031).
