# Supplementary material for: Longitudinal predictors of weapon involvement in middle adolescence: Evidence from the UK Millennium Cohort Study
Source: Aggress Behav. 2022 Aug 17;49(1):5–14. doi: 10.1002/ab.22049 (PMC10087410; doi:10.1002/ab.22049)
Supplement: Supplementary file 1 — Supporting information. [file AB-49-5-s001.docx]

**Supporting information**

**Table S1** Measurement of predictor variables

| **Sex at birth**: Reported by the main parent in the initial survey  **Age at age 17:** Cohort member’s age in months at the date of the interview, derived from interview date and birthdate of the cohort member.  **Eldest child in household:** Derived a household questionnaire with the main parent collecting information on age, sex, and relationship of household members to the cohort member.  **Ethnicity**: Ethnicity of the cohort member. Originally reported by the main parent in the initial survey using 16 categories. The variables used in the current study is a condensed version using 6 categories. |
| --- |
| **Household income weekly (average 9 months to age 11)**:  Household income was reported by the main parent shown a card with weekly, monthly, and annual bands of income. Based on these bands a continuous income measure was estimated using relevant predictor variables. Finally, income was equivalised using modified OECD scales, which takes account of the household size and composition, thereby factoring in the needs of the family. |
| **Breastfed ever:** The main parent reported in the initial survey whether the child had ever been breastfed.  **Mother smoked during pregnancy after second month:** The main parent (predominately the mother) reported their smoking habits prior to and during the pregnancy, including month of cessation (if the mother’s partner completed the main parent interview, then they reported on the mother’s smoking during pregnancy. Smoking during pregnancy was defined as smoking after the second month of pregnancy. Anyone giving up before then were classified as not smoking during pregnancy.  **Age 3: Parent-child relationship (parent reported)**  Parent completed the Pianta Child-Parent Relationship Scale, 15-item short form which measures both parent-child closeness and parent-child conflict.^1^ |
| **Main parent mental health problems (9mths-11yrs)**  The main respondent completed the Malaise in the initial birth sweep and in subsequent sweeps at age 3, 5, 7, and 11 the Kessler was used to assess mental health.^2^ A composite measure was then created combining parental mental health across childhood.  **Domestic abuse between parents (9mths-11yrs)**  In all five sweeps from birth to age 11, the main respondent and the partner (where available), were asked: ‘People often use force in a relationship - grabbing, pushing, shaking, hitting, kicking etc. Has your partner ever used force on you for any reason? [yes/no]  If either party responded yes at any sweep this was counted as domestic abuse.  **Main parent used recreational drugs age 3,5, or 14**  The main parent reporting use of recreational drugs at age 3, 5 and 14 through the question: ‘As you know many people have experimented with drugs at some time. During the past year have you used any recreational drugs like cannabis, cocaine or  ecstasy?’ (Occasionally, Regularly, Never). Occasional or regular use was counted as having used recreational drugs).  **Ever a single parent between 9mths and 11yrs**  The household survey recorded who lived in the household and their relationship to the main respondent. If the main respondent in any sweep between the birth sweep to age 11 reported no partner living with them this was considered a single parent. |
| **Academic achievement at age 16 (Five or more GCSEs grade C or above)**  Participants self-reported their GCSE results at age 17  **School exclusion in secondary between age 11 and 14**  Parents reported both at age 11 and at age 14:  ‘Has [cohort member's name] ever been temporarily suspended or temporarily excluded from school for at least one day?’ (yes, no)  **Persistent truancy (more than just the once) past year at age 14**  Self-reported at age 14 by asking participants: ‘In the last 12 months, how often did you miss school without your parents’ permission (even if only for half a day or a single lesson)? (Most days, 2-3 times a week, Once a week, Once a month, Less than once a month, Once) |
| **Age 14: Time spent with friends in leisure time**  In the afternoon after school, how often do you spend time with your friends, but without adults or older children, doing things like playing in the park, going to the shops or just ‘hanging out’? (Most days, At least once a week, At least once a month, Less often than once a month, Never)  **Age 14: Victim of peer bullying**  How often do other children hurt you or pick on you on purpose? (Most days, Once a week, Once a month, Every few months, Less often, Never)  (A binary measure was created for the regression analyses: No=Never, Yes= Most days, Once a week, Once a month, Every few months, Less often)  **Peer substance use:**  Age 14: Friends smoke cigarettes  How many of your friends smoke cigarettes? Do not include electronic cigarettes (e-cigarettes). (None of them, Some of them, Most of them, All of them)  (Binary measure created for correlational analyses: No=Never, Yes=Some, most, or all of them)  Age 14: Friends drink alcohol  How many of your friends drink alcohol? (None of them, Some of them, Most of them, All of them)  (Binary measure created for correlational analyses: No=Never, Yes=Some, most, or all of them)  Age 14: Friends take drugs  Do any of your friends take cannabis (weed) or any other illegal drugs? (None of them, Some of them, Most of them, All of them)  (Binary measure created for correlational analyses: No=Never, Yes=Some, most, or all of them)  For regressions a single measure of peer substance use was created:  None, One type of substance, Two or three types of substances |
| **Childhood externalising problems (age 3-11):** Measured through parent-reports using 10 items from the Strengths and Difficulties Questionnaire (SDQ).^4^  Five items relate to conduct problems (Often has temper tantrums or hot tempers; Generally obedient, usually does what adults request; Often fights with other children or bullies them; Often lies or cheats; Steals from home, school or elsewhere.).  Five items are on hyperactivity (Restless, overactive, cannot stay still for long; Constantly fidgeting or squirming; Easily distracted, concentration wanders; Thinks things out before acting; Sees tasks through to the end, good attention span).  **Childhood internalising problems (age 3-11):** Measured through parent-reports using 10 items from the Strengths and Difficulties Questionnaire (SDQ). ^4^  Five items assess emotional problems (Often complains of headaches, stomach-aches or sickness; Many worries, often seems worried; Often unhappy, down-hearted or tearful; Nervous or clingy in new situations, easily loses confidence; Many fears, easily scared).  Five items measure peer problems (Rather solitary, tends to play alone; Has at least one good friend; Generally liked by other children; Picked on or bullied by other children; Gets on better with adults than with other children).  **Age 14: Self-harm in past year:** Measured in a single question (In the past year have you hurt yourself on purpose in any way?) |
| **Age 14: Social media time use per weekday**  On a normal week day during term time, how many hours do you spend on social networking or messaging sites or Apps on the internet such as Facebook, Twitter and WhatsApp?  (None, Less than half an hour, Half an hour to less than 1 hour, 1 hour to less than 2 hours, 2 hours to less than 3 hours, 3 hours to less than 5 hours, 5 hours to less than 7 hours, 7 hours or more)  The categorical variable was transformed into ridit scores for use in regressions.^5^  **Age 14: Computer/electronic gaming time use per weekday**  On a normal week day during term time, how many hours do you spend playing electronic games on a computer or games systems, such as Wii, Nintendo D-S, X-Box or PlayStation? Please remember to include time before school as well as time after school.  (None, Less than half an hour, Half an hour to less than 1 hour, 1 hour to less than 2 hours, 2 hours to less than 3 hours, 3 hours to less than 5 hours, 5 hours to less than 7 hours, 7 hours or more). The ordered categorical variable was transformed into ridit scores for use in regressions.^5^ |
| **Substance use**  Binge drinking in past year: How many times have you had five or more alcoholic drinks at a time in the last 12 months?  Regular smoker: ‘I usually smoke between one and six cigarettes a week.’ ‘I usually smoke more than six cigarettes a week.’  Tried cannabis ever: Have you ever tried cannabis (also known as weed, marijuana, dope, hash or skunk)?  Tried hard drugs ever: Have you ever tried any other illegal drug (such as ecstasy, cocaine, speed)?  For regressions a single measure of substance use was created:  (None, One type of substance, Two or three types of substances) For this single measure cannabis and drugs were here combined as ‘drugs’, the other types were binge drinking and regular smoking) |
| *Notes*: Table references  Driscoll, K., & Pianta, R. C. (2011). Mothers' and fathers' perceptions of conflict and closeness in parent-child relationships during early childhood. Journal of Early Childhood and Infant Psychology, (7), 1-24.  2 Rutter, M., Tizard, J., & Whitmore, K. (1970). Education, health and behaviour: Longman Publishing Group.  3 Kessler, R. C., Barker, P. R., Colpe, L. J., Epstein, J. F., Gfroerer, J. C., Hiripi, E., . . . Zaslavsky, A. M. (2003). Screening for serious mental illness in the general population. Arch Gen Psychiatry, 60(2), 184-189.  4 Goodman, R. (1997). The Strengths and Difficulties Questionnaire: a research note. *Journal of child psychology and psychiatry*, *38*(5), 581-586.  5 Jansen, M. E. (1984). Ridit analysis, a review. Statistica Neerlandica, 38(3), 141-158. |

**Table S2** Results of multivariate logistic regression predicting weapon involvement at age 17 (Males: N=6,708)

|  | Model 1 |  | Model 2 |  | Model 3 |  |
| --- | --- | --- | --- | --- | --- | --- |
|  | OR | 95% CI | OR | 95% CI | OR | 95% CI |
| **INDIVIDUAL CHARACTERISTICS** |  |  |  |  |  |  |
| Eldest child in household | 0.96 | (0.68 - 1.33) | 0.98 | (0.71 - 1.37) | 1.06 | (0.76 - 1.48) |
| Cohort member age in months at age 17 survey | 1.00 | (0.96 - 1.04) | 1.00 | (0.96 - 1.04) | 0.99 | (0.95 - 1.03) |
| Ethnicity (ref. White) |  |  |  |  |  |  |
| Mixed | 0.85 | (0.36 - 2.0) | 0.88 | (0.37 - 2.05) | 0.93 | (0.38 - 2.30) |
| Indian | 0.83 | (0.28 - 2.45) | 0.90 | (0.30 - 2.71) | 1.37 | (0.47 - 4.02) |
| Pakistani and Bangladeshi | 0.74 | (0.44 - 1.25) | 0.85 | (0.49 - 1.47) | 1.28 | (0.73 - 2.26) |
| Black or Black British | 0.77 | (0.32 - 1.83) | 0.84 | (0.36 - 1.98) | 1.09 | (0.46 - 2.60) |
| Other Ethnic group (incl. Chinese) | 0.55 | (0.10 - 3.18) | 0.61 | (0.11 - 3.52) | 0.80 | (0.13 - 4.73) |
| **FAMILY SOCIOECONOMICS** |  |  |  |  |  |  |
| Household income weekly (average 9mths to age 11) (ref. 80-100% highest) | | |  |  |  |  |
| 20% lowest | 1.67+ | (0.92 - 3.03) | 1.53 | (0.84 - 2.80) | 1.30 | (0.70 - 2.41) |
| 20-40% | 1.46 | (0.85 - 2.50) | 1.38 | (0.81 - 2.37) | 1.20 | (0.70 - 2.07) |
| 40-60% | 1.23 | (0.77 - 1.96) | 1.18 | (0.74 - 1.89) | 1.11 | (0.69 - 1.79) |
| 60-80% highest | 1.20 | (0.76 - 1.90) | 1.17 | (0.74 - 1.85) | 1.13 | (0.71 - 1.79) |
| **FAMILY ENVIRONMENT** |  |  |  |  |  |  |
| Breastfed | 0.89 | (0.64 - 1.25) | 0.91 | (0.65 - 1.28) | 0.93 | (0.66 - 1.32) |
| Mother smoked during pregnancy | 1.19 | (0.82 - 1.72) | 1.14 | (0.79 - 1.66) | 1.01 | (0.68 - 1.51) |
| Parent-child relationship (parent reported) age 3 ^a^ | 0.95 | (0.82 - 1.11) | 1.05 | (0.88 - 1.25) | 1.03 | (0.86 - 1.22) |
| Main parent mental health problems (9mths-11yrs) ^a^ | 1.07 | (0.92 - 1.24) | 1.06 | (0.90 - 1.24) | 1.06 | (0.90 - 1.24) |
| Domestic abuse between parents (9mths-11yrs) | **1.39*** | (1.01 - 1.92) | 1.36+ | (0.98 - 1.90) | 1.25 | (0.89 - 1.76) |
| Main parent used recreational drugs (age 3,5 or 14) | 1.37 | (0.85 - 2.19) | 1.34 | (0.82 - 2.19) | 1.10 | (0.65 - 1.86) |
| Single parent ever between 9mths and 11yrs | 1.36 | (0.94 - 1.95) | 1.33 | (0.92 - 1.92) | 1.11 | (0.76 - 1.62) |
| **MENTAL HEALTH** | |  |  |  |  |  |
| Childhood externalising problems (age 3-11) ^a^ |  |  | **1.32**** | (1.08 - 1.60) | 1.10 | (0.91 - 1.34) |
| Childhood internalising problems (age 3-11) ^a^ |  |  | 0.86 | (0.70 - 1.05) | 0.92 | (0.75 - 1.14) |
| Age 14: Self-harmed in past year |  |  | **1.99**** | (1.30 - 3.06) | 1.29 | (0.81 - 2.04) |
| **SUBSTANCE USE AT AGE 14** |  |  |  |  |  |  |
| Binge drinking, regular smoking, trying cannabis/drugs (ref. none of these) | | | |  |  |  |
| One type of substance |  |  |  |  | **1.62*** | (1.00 - 2.63) |
| Two or three types of substances |  |  |  |  | **2.16*** | (1.06 - 4.39) |
| **SOCIAL MEDIA AND GAMING AT AGE 14** |  |  |  |  |  |  |
| Social media time use ^b^ |  |  |  |  | 1.09 | (0.58 - 2.05) |
| Computer/electronic gaming time use ^b^ |  |  |  |  | 1.78+ | (0.91 - 3.47) |
| **SCHOOL FACTORS** |  |  |  |  |  |  |
| Academic achievement at age 16 (Five GCSEs grade C or above) | |  |  |  | 0.80 | (0.58 - 1.09) |
| School exclusion in secondary between age 11 and 14 |  |  |  |  | **1.71*** | (1.02 - 2.86) |
| Persistent truancy (more than just the once) in past year at age 14 | |  |  |  | 1.48 | (0.85 - 2.58) |
| **PEER FACTORS AT AGE 14** |  |  |  |  |  |  |
| Spending time with friends in leisure time on most days | |  |  |  | 1.11 | (0.83 - 1.48) |
| Victim of peer bullying |  |  |  |  | 1.04 | (0.77 - 1.40) |
| Peer substance use (alcohol, smoking, drugs) (ref. no substance use) | | | | | |  |
| One type of substance |  |  |  |  | 1.48+ | (0.93 - 2.37) |
| Two or three types of substances |  |  |  |  | **2.22***** | (1.45 - 3.39) |
| NOTES:  *** p<0.001, ** p<0.01, * p<0.05, + p<0.10  OR=Odds Ratio  ^a^ This predictor variable is standardised (z score), meaning that the odds ratio coefficient is for one standard deviation increase in the predictor.  ^b^ This predictor variable is a ridit score, which is a transformation of ordinal scale responses (hourly time-use bands) into a continuous measure. The odds ratio coefficient corresponds to differences between those with the highest time use (7 hours or more) compared to those with the lowest (no time spent). | | | | | | |

**Table S3** Results of multivariate logistic regression predicting weapon involvement at age 17 (Females: N=6,569)

|  | Model 1 |  | Model 2 |  | Model 3 |  |
| --- | --- | --- | --- | --- | --- | --- |
|  | OR | 95% CI | OR | 95% CI | OR | 95% CI |
| **INDIVIDUAL CHARACTERISTICS** |  |  |  |  |  |  |
| Eldest child in household | 0.85 | (0.53 - 1.37) | 0.86 | (0.53 - 1.40) | 0.92 | (0.57 - 1.48) |
| Cohort member age in months at age 17 survey | 0.99 | (0.94 - 1.05) | 1.00 | (0.94 - 1.05) | 0.98 | (0.93 - 1.04) |
| Ethnicity (ref. White) |  |  |  |  |  |  |
| Mixed | 1.02 | (0.35 - 2.95) | 1.02 | (0.34 - 3.03) | 1.06 | (0.34 - 3.30) |
| Indian | 0.52 | (0.09 - 3.17) | 0.59 | (0.10 - 3.58) | 0.80 | (0.13 - 4.87) |
| Pakistani and Bangladeshi | 0.64 | (0.26 - 1.63) | 0.81 | (0.32 - 2.04) | 1.12 | (0.41 - 3.04) |
| Black or Black British | 0.71 | (0.20 - 2.53) | 0.93 | (0.26 - 3.30) | 1.20 | (0.35 - 4.16) |
| Other Ethnic group (incl. Chinese) | 0.38 | (0.03 - 5.76) | 0.45 | (0.03 - 6.89) | 0.65 | (0.04 - 9.89) |
| **FAMILY SOCIOECONOMICS** |  |  |  |  |  |  |
| Household income weekly (average 9mths to age 11) (ref. 80-100% highest) | | |  |  |  |  |
| 20% lowest | 1.84 | (0.80 - 4.30) | 1.63 | (0.68 - 3.95) | 1.37 | (0.55 - 3.41) |
| 20-40% | 1.80 | (0.89 - 3.64) | 1.62 | (0.79 - 3.33) | 1.51 | (0.72 - 3.14) |
| 40-60% | 1.22 | (0.59 - 2.51) | 1.15 | (0.56 - 2.36) | 1.11 | (0.54 - 2.32) |
| 60-80% highest | 1.15 | (0.56 - 2.38) | 1.11 | (0.53 - 2.31) | 1.09 | (0.52 - 2.31) |
| **FAMILY ENVIRONMENT** |  |  |  |  |  |  |
| Breastfed | 0.90 | (0.59 - 1.40) | 0.89 | (0.58 - 1.36) | 0.95 | (0.61 - 1.47) |
| Mother smoked during pregnancy | 1.38 | (0.82 - 2.33) | 1.34 | (0.80 - 2.24) | 1.17 | (0.68 - 2.03) |
| Parent-child relationship (parent reported) age 3 ^a^ | 0.95 | (0.74 - 1.22) | 1.03 | (0.78 - 1.35) | 1.00 | (0.76 - 1.32) |
| Main parent mental health problems (9mths-11yrs) ^a^ | 1.03 | (0.80 - 1.34) | 1.01 | (0.76 - 1.33) | 1.01 | (0.76 - 1.34) |
| Domestic abuse between parents (9mths-11yrs) | 1.30 | (0.79 - 2.13) | 1.29 | (0.78 - 2.11) | 1.21 | (0.72 - 2.04) |
| Main parent used recreational drugs (age 3,5 or 14) | 1.67 | (0.80 - 3.48) | 1.55 | (0.74 - 3.24) | 1.31 | (0.60 - 2.87) |
| Single parent ever between 9mths and 11yrs | 1.19 | (0.72 - 1.97) | 1.13 | (0.68 - 1.87) | 1.02 | (0.61 - 1.69) |
| **MENTAL HEALTH** | |  |  |  |  |  |
| Childhood externalising problems (age 3-11) ^a^ |  |  | 1.24 | (0.95 - 1.63) | 1.08 | (0.80 - 1.44) |
| Childhood internalising problems (age 3-11) ^a^ |  |  | 0.90 | (0.69 - 1.19) | 0.92 | (0.69 - 1.23) |
| Age 14: Self-harmed in past year |  |  | **2.20***** | (1.42 - 3.39) | 1.52 | (0.90 - 2.54) |
| **SUBSTANCE USE AT AGE 14** |  |  |  |  |  |  |
| Binge drinking, regular smoking, trying cannabis/drugs (ref. none of these) | | | |  |  |  |
| One type of substance |  |  |  |  | 1.20 | (0.58 - 2.49) |
| Two or three types of substances |  |  |  |  | 1.87 | (0.86 - 4.08) |
| **SOCIAL MEDIA AND GAMING AT AGE 14** |  |  |  |  |  |  |
| Social media time use ^b^ |  |  |  |  | 1.06 | (0.41 - 2.77) |
| Computer/electronic gaming time use ^b^ |  |  |  |  | 1.64 | (0.67 - 3.98) |
| **SCHOOL FACTORS** |  |  |  |  |  |  |
| Academic achievement at age 16 (Five GCSEs grade C or above) | |  |  |  | 0.84 | (0.52 - 1.37) |
| School exclusion in secondary between age 11 and 14 |  |  |  |  | 2.00 | (0.80 - 4.99) |
| Persistent truancy (more than just the once) in past year at age 14 | |  |  |  | 1.36 | (0.56 - 3.33) |
| **PEER FACTORS AT AGE 14** |  |  |  |  |  |  |
| Spending time with friends in leisure time on most days | |  |  |  | 1.27 | (0.77 - 2.08) |
| Victim of peer bullying |  |  |  |  | 1.38 | (0.86 - 2.20) |
| Peer substance use (alcohol, smoking, drugs) (ref. no substance use) | | | | | |  |
| One type of substance |  |  |  |  | 1.08 | (0.58 - 2.02) |
| Two or three types of substances |  |  |  |  | 1.53 | (0.81 - 2.92) |
| NOTES:  *** p<0.001, ** p<0.01, * p<0.05, + p<0.10  OR=Odds Ratio  ^a^ This predictor variable is standardised (z score), meaning that the odds ratio coefficient is for one standard deviation increase in the predictor.  ^b^ This predictor variable is a ridit score, which is a transformation of ordinal scale responses (hourly time-use bands) into a continuous measure. The odds ratio coefficient corresponds to differences between those with the highest time use (7 hours or more) compared to those with the lowest (no time spent). | | | | | | |

**Table S4:** Results of multivariate logistic regression predicting weapon involvement at age 17 (with interaction for sex on predictors significant in main model)

|  | Model 1 |  | Model 2 |  | Model 3 |  |
| --- | --- | --- | --- | --- | --- | --- |
|  | OR | 95% CI | OR | 95% CI | OR | 95% CI |
| **INDIVIDUAL CHARACTERISTICS** |  |  |  |  |  |  |
| Male | **2.50**** | (1.40 - 4.46) | **2.69**** | (1.47 - 4.93) | 1.77 | (0.71 - 4.39) |
| Eldest child in household | 0.93 | (0.70 - 1.23) | 0.95 | (0.71 - 1.26) | 1.01 | (0.76 - 1.35) |
| Cohort member age in months at age 17 survey | 1.00 | (0.96 - 1.03) | 1.00 | (0.97 - 1.03) | 0.99 | (0.95 - 1.02) |
| Ethnicity (ref. White) |  |  |  |  |  |  |
| Mixed | 0.91 | (0.45 - 1.83) | 0.93 | (0.46 - 1.88) | 0.98 | (0.46 - 2.09) |
| Indian | 0.78 | (0.30 - 2.01) | 0.85 | (0.33 - 2.22) | 1.27 | (0.50 - 3.23) |
| Pakistani and Bangladeshi | 0.71 | (0.45 - 1.12) | 0.84 | (0.52 - 1.35) | 1.24 | (0.77 - 1.99) |
| Black or Black British | 0.76 | (0.36 - 1.62) | 0.88 | (0.41 - 1.85) | 1.14 | (0.55 - 2.36) |
| Other Ethnic group (incl. Chinese) | 0.55 | (0.12 - 2.59) | 0.63 | (0.13 - 2.95) | 0.85 | (0.18 - 4.078) |
| **FAMILY SOCIOECONOMICS** |  |  |  |  |  |  |
| Household income weekly (average 9mths to age 11) (ref. 80-100% highest) | | |  |  |  |  |
| 20% lowest | 1.81 | (0.82 - 4.009) | 1.64 | (0.74 - 3.66) | 1.33 | (0.58 - 3.04) |
| 20-40% | 1.78+ | (0.90 - 3.53) | 1.62 | (0.82 - 3.21) | 1.48 | (0.74 - 2.97) |
| 40-60% | 1.21 | (0.60 - 2.45) | 1.15 | (0.57 - 2.31) | 1.10 | (0.54 - 2.25) |
| 60-80% highest | 1.14 | (0.56 - 2.35) | 1.11 | (0.54 - 2.28) | 1.09 | (0.52 - 2.28) |
| 20% lowest x male | 0.94 | (0.41 - 2.12) | 0.94 | (0.41 - 2.160) | 1.00 | (0.41 - 2.41) |
| 20-40% x male | 0.83 | (0.39 - 1.77) | 0.86 | (0.40 - 1.89) | 0.82 | (0.37 - 1.81) |
| 40-60% x male | 1.02 | (0.43 - 2.44) | 1.03 | (0.44 - 2.44) | 1.01 | (0.42 - 2.41) |
| 60-80% highest x male | 1.05 | (0.49 - 2.28) | 1.06 | (0.49 - 2.30) | 1.04 | (0.47 - 2.31) |
| **FAMILY ENVIRONMENT** |  |  |  |  |  |  |
| Breastfed | 0.89 | (0.69 - 1.15) | 0.90 | (0.70 - 1.16) | 0.93 | (0.72 - 1.21) |
| Mother smoked during pregnancy | 1.25 | (0.91 - 1.71) | 1.20 | (0.88 - 1.64) | 1.06 | (0.76 - 1.48) |
| Parent-child relationship (parent reported) age 3 ^a^ | 0.95 | (0.84 - 1.09) | 1.04 | (0.90 - 1.21) | 1.02 | (0.88 - 1.18) |
| Main parent mental health problems (9mths-11yrs) ^a^ | 1.06 | (0.93 - 1.21) | 1.04 | (0.90 - 1.20) | 1.04 | (0.90 - 1.21) |
| Domestic abuse between parents (9mths-11yrs) | 1.33 | (0.83 - 2.13) | 1.30 | (0.81 - 2.10) | 1.23 | (0.74 - 2.04) |
| Domestic abuse between parents (9mths-11yrs) x male | 1.03 | (0.60 - 1.79) | 1.04 | (0.59 - 1.82) | 1.01 | (0.56 - 1.81) |
| Main parent used recreational drugs (age 3,5 or 14) | 1.46+ | (0.95 - 2.27) | 1.41 | (0.90 - 2.21) | 1.17 | (0.72 - 1.90) |
| Single parent ever between 9mths and 11yrs | 1.30 | (0.95 - 1.78) | 1.26 | (0.92 - 1.73) | 1.08 | (0.78 - 1.50) |
| **MENTAL HEALTH** | |  |  |  |  |  |
| Childhood externalising problems (age 3-11) ^a^ |  |  | 1.26+ | (0.98 - 1.62) | 1.08 | (0.83 - 1.40) |
| Childhood externalising problems (age 3-11) x male |  |  | 1.04 | (0.79 - 1.36) | 1.02 | (0.78 - 1.34) |
| Childhood internalising problems (age 3-11) ^a^ |  |  | 0.87 | (0.74 - 1.03) | 0.92 | (0.78 - 1.10) |
| Age 14: Self-harmed in past year |  |  | **2.20***** | (1.41 - 3.42) | 1.58+ | (0.97 - 2.58) |
| Age 14: Self-harmed in past year x male |  |  | 0.90 | (0.47 - 1.73) | 0.80 | (0.40 - 1.60) |
| **SUBSTANCE USE AT AGE 14** |  |  |  |  |  |  |
| Binge drinking, regular smoking, trying cannabis/drugs (ref. none of these) | | | |  |  |  |
| One type of substance |  |  |  |  | 1.24 | (0.61 - 2.54) |
| Two or three types of substances |  |  |  |  | 1.92+ | (0.90 - 4.07) |
| One type of substance x male |  |  |  |  | 1.30 | (0.54 - 3.12) |
| Two or three types of substances x male |  |  |  |  | 1.12 | (0.44 - 2.86) |
| **SOCIAL MEDIA AND GAMING AT AGE 14** |  |  |  |  |  |  |
| Social media time use ^b^ |  |  |  |  | 1.08 | (0.623 - 1.86) |
| Computer/electronic gaming time use ^b^ |  |  |  |  | 1.69 | (0.71 - 4.05) |
| Computer/electronic gaming time use x male |  |  |  |  | 1.05 | (0.36 - 3.08) |
| **SCHOOL FACTORS** |  |  |  |  |  |  |
| Academic achievement at age 16 (Five GCSEs grade C or above) | |  |  |  | 0.81 | (0.61 - 1.07) |
| School exclusion in secondary between age 11 and 14 |  |  |  |  | 1.97 | (0.80 - 4.85) |
| School exclusion in secondary between age 11 and 14 x male |  |  |  |  | 0.86 | (0.36 - 2.09) |
| Persistent truancy (more than just the once) in past year at age 14 | |  |  |  | 1.43 | (0.89 - 2.30) |
| **PEER FACTORS AT AGE 14** |  |  |  |  |  |  |
| Spending time with friends in leisure time on most days | |  |  |  | 1.16 | (0.89 - 1.50) |
| Victim of peer bullying |  |  |  |  | 1.13 | (0.88 - 1.45) |
| Peer substance use (alcohol, smoking, drugs) (ref. no substance use) | | | | | |  |
| One type of substance |  |  |  |  | 1.12 | (0.61 - 2.04) |
| Two or three types of substances |  |  |  |  | 1.59 | (0.84 - 3.02) |
| One type of substance x male |  |  |  |  | 1.31 | (0.60 - 2.84) |
| Two or three types of substances x male |  |  |  |  | 1.36 | (0.66 - 2.82) |
| NOTES:  *** p<0.001, ** p<0.01, * p<0.05, + p<0.10  OR=Odds Ratio  ^a^ This predictor variable is standardised (z score), meaning that the odds ratio coefficient is for one standard deviation increase in the predictor.  ^b^ This predictor variable is a ridit score, which is a transformation of ordinal scale responses (hourly time-use bands) into a continuous measure. The odds ratio coefficient corresponds to differences between those with the highest time use (7 hours or more) compared to those with the lowest (no time spent). | | | | | | |

**Table S5:** Results of multivariate logistic regression predicting weapon involvement at age 17 (excluding childhood externalising as predictor)

|  | Model 1 |  | Model 2 |  | Model 3 |  |
| --- | --- | --- | --- | --- | --- | --- |
|  | OR | 95% CI | OR | 95% CI | OR | 95% CI |
| **INDIVIDUAL CHARACTERISTICS** |  |  |  |  |  |  |
| Male | **2.36***** | (1.86 - 3.02) | **2.76***** | (2.17 - 3.59) | **2.15***** | (1.53 - 3.01) |
| Eldest child in household | 0.93 | (0.70 - 1.231) | 0.94 | (0.70 - 1.25) | 1.01 | (0.76 - 1.35) |
| Cohort member age in months at age 17 survey | 1.00 | (0.96 - 1.03) | 1.00 | (0.96 - 1.03) | 0.98 | (0.95 - 1.02) |
| Ethnicity (ref. White) |  |  |  |  |  |  |
| Mixed | 0.91 | (0.45 - 1.83) | 0.92 | (0.46 - 1.85) | 0.98 | (0.46 - 2.06) |
| Indian | 0.78 | (0.30 - 2.02) | 0.84 | (0.32 - 2.18) | 1.28 | (0.50 - 3.26) |
| Pakistani and Bangladeshi | 0.71 | (0.45 - 1.12) | 0.79 | (0.49 - 1.27) | 1.24 | (0.77 - 2.01) |
| Black or Black British | 0.76 | (0.36 - 1.61) | 0.83 | (0.39 - 1.75) | 1.11 | (0.54 - 2.32) |
| Other Ethnic group (incl. Chinese) | 0.55 | (0.12 - 2.58) | 0.58 | (0.12 - 2.73) | 0.85 | (0.18 - 4.11) |
| **FAMILY SOCIOECONOMICS** |  |  |  |  |  |  |
| Household income weekly (average 9mths to age 11) (ref. 80-100% highest) | | |  |  |  |  |
| 20% lowest | **1.73*** | (1.03 - 2.90) | **1.73*** | (1.03 - 2.90) | 1.35 | (0.80 - 2.29) |
| 20-40% | 1.57+ | (0.99 - 2.47) | 1.55+ | (0.98 - 2.45) | 1.32 | (0.84 - 2.08) |
| 40-60% | 1.23 | (0.85 - 1.79) | 1.22 | (0.84 - 1.79) | 1.13 | (0.77 - 1.66) |
| 60-80% highest | 1.19 | (0.78 - 1.81) | 1.19 | (0.78 - 1.81) | 1.13 | (0.74 - 1.73) |
| **FAMILY ENVIRONMENT** |  |  |  |  |  |  |
| Breastfed | 0.89 | (0.69 - 1.15) | 0.87 | (0.68 - 1.12) | 0.93 | (0.72 - 1.20) |
| Mother smoked during pregnancy | 1.25 | (0.91 - 1.71) | 1.23 | (0.90 - 1.68) | 1.07 | (0.77 - 1.49) |
| Parent-child relationship (parent reported) age 3 ^a^ | 0.95 | (0.84 - 1.09) | 0.95 | (0.83 - 1.08) | 0.98 | (0.86 - 1.13) |
| Main parent mental health problems (9mths-11yrs) ^a^ | 1.06 | (0.92 - 1.21) | 1.06 | (0.92 - 1.22) | 1.05 | (0.91 - 1.21) |
| Domestic abuse between parents (9mths-11yrs) | **1.36*** | (1.04 - 1.80) | **1.36*** | (1.03 - 1.79) | 1.24 | (0.93 - 1.66) |
| Main parent used recreational drugs (age 3,5 or 14) | 1.47+ | (0.95 - 2.28) | 1.40 | (0.90 - 2.17) | 1.16 | (0.72 - 1.88) |
| Single parent ever between 9mths and 11yrs | 1.29 | (0.95 - 1.77) | 1.28 | (0.93 - 1.76) | 1.08 | (0.78 - 1.51) |
| **MENTAL HEALTH** | |  |  |  |  |  |
| Childhood internalising problems (age 3-11) ^a^ |  |  | 0.94 | (0.81 - 1.10) | 0.95 | (0.80 - 1.12) |
| Age 14: Self-harmed in past year |  |  | **2.16***** | (1.62 - 2.87) | 1.39+ | (0.99 - 1.94) |
| **SUBSTANCE USE AT AGE 14** |  |  |  |  |  |  |
| Binge drinking, regular smoking, trying cannabis/drugs (ref. none of these) | | | |  |  |  |
| One type of substance |  |  |  |  | **1.50*** | (1.03 - 2.20) |
| Two or three types of substances |  |  |  |  | **2.10**** | (1.22 - 3.62) |
| **SOCIAL MEDIA AND GAMING AT AGE 14** |  |  |  |  |  |  |
| Social media time use ^b^ |  |  |  |  | 1.09 | (0.63 - 1.88) |
| Computer/electronic gaming time use ^b^ |  |  |  |  | **1.78*** | (1.04 - 3.06) |
| **SCHOOL FACTORS** |  |  |  |  |  |  |
| Academic achievement at age 16 (Five GCSEs grade C or above) | |  |  |  | 0.79+ | (0.60 - 1.03) |
| School exclusion in secondary between age 11 and 14 |  |  |  |  | **1.83*** | (1.12 - 2.99) |
| Persistent truancy (more than just the once) in past year at age 14 | |  |  |  | 1.42 | (0.89 - 2.27) |
| **PEER FACTORS AT AGE 14** |  |  |  |  |  |  |
| Spending time with friends in leisure time on most days | |  |  |  | 1.16 | (0.89 - 1.50) |
| Victim of peer bullying |  |  |  |  | 1.14 | (0.89 - 1.46) |
| Peer substance use (alcohol, smoking, drugs) (ref. no substance use) | | | | | |  |
| One type of substance |  |  |  |  | 1.37+ | (0.95 - 1.98) |
| Two or three types of substances |  |  |  |  | **2.01***** | (1.40 - 2.88) |
| NOTES:  *** p<0.001, ** p<0.01, * p<0.05, + p<0.10  OR=Odds Ratio  ^a^ This predictor variable is standardised (z score), meaning that the odds ratio coefficient is for one standard deviation increase in the predictor.  ^b^ This predictor variable is a ridit score, which is a transformation of ordinal scale responses (hourly time-use bands) into a continuous measure. The odds ratio coefficient corresponds to differences between those with the highest time use (7 hours or more) compared to those with the lowest (no time spent). | | | | | | |
